# Development of the gas chromatography/mass spectrometry-based aroma designer capable of modifying volatile chemical compositions in complex odors

**DOI:** 10.1093/chemse/bjae007

**Published:** 2024-02-22

**Authors:** Kaname Obara, Reiko Uenoyama, Yutaro Obata, Masao Miyazaki

**Affiliations:** Division of Agriculture, Graduate School of Arts and Sciences, Iwate University, 3-18-8 Ueda, Morioka, Iwate 020-8550, Japan; Department of Bioresources Science, The United Graduate School of Agricultural Sciences, Iwate University, 3-18-8 Ueda, Morioka, Iwate 020-8550, Japan; Department of Biological Chemistry and Food Sciences, Faculty of Agriculture, Iwate University, 3-18-8 Ueda, Morioka, Iwate 020-8550, Japan; Division of Agriculture, Graduate School of Arts and Sciences, Iwate University, 3-18-8 Ueda, Morioka, Iwate 020-8550, Japan; Department of Bioresources Science, The United Graduate School of Agricultural Sciences, Iwate University, 3-18-8 Ueda, Morioka, Iwate 020-8550, Japan; Department of Biological Chemistry and Food Sciences, Faculty of Agriculture, Iwate University, 3-18-8 Ueda, Morioka, Iwate 020-8550, Japan

**Keywords:** gas chromatography/mass spectrometry, omission test, volatile organic compound, aroma, key odorants

## Abstract

Many volatile organic compounds (VOCs) are used to produce various commercial products with aromas mimicking natural products. The VOCs responsible for aromas have been identified from many natural products. The current major strategy is to analyze chemical compositions and aroma qualities of individual VOCs using gas chromatography/mass spectrometry (GC/MS) and GC-olfactometry. However, such analyses cannot determine whether candidate VOCs contribute to the characteristic aroma in mixtures of many VOCs. In this study, we developed a GC/MS-based VOC collection/omission system that can modify the VOC compositions of samples easily and rapidly. The system is composed of GC/MS with a switching unit that can change gas flow routes between MS and a VOC collection device. We first applied this system to prepare gas samples for omission tests, and the aroma qualities of VOC mixtures with and without some VOCs were evaluated by panelists. If aroma qualities were different between the 2 samples, the omitted VOCs were likely key odorants. By collecting VOCs in a gas bag attached to the collection device and transferring some VOCs to MS, specific VOCs could be omitted easily from the VOC mixture. The system could prepare omission samples without chemical identification, preparation of each VOC, and laborious techniques for mixing VOCs, thus overcoming the limitations of previous methods of sample preparation. Finally, the system was used to prepare artificial aromas by replacing VOC compositions between different samples for screening of key odorants. In conclusion, the system developed here can improve aroma research by identifying key odorants from natural products.

## Introduction

Various commercial products, including beverages, foodstuffs, cosmetics, detergents, and deodorants, are developed by imitating the aromas of natural products, such as flowers, fruits, leaves, and bark. To develop these products at low cost and on large scales, volatile organic compounds (VOCs) identified from the natural products are chemically synthesized as flavor and fragrance rather than using extracts of these products (Rowe 2009). Natural products have complex compositions of VOCs, which are sensed as a wide variety of odors in the human olfactory system (Buck 2004; Block 2018). However, not all VOCs are involved in the formation of odors (Acree et al. 1984). There are VOCs whose presence makes little contribution to human sensing (Grosch 1993). Therefore, the identification of key VOCs from natural products is required for the development of flavors and fragrances for commercial products.

Gas chromatography/mass spectrometry (GC/MS) is a gold standard for comprehensive qualitative and quantitative analyses of VOCs emitted from natural products (Baldovini and Chaintreau 2020). In addition, the GC-olfactometry (GC-O) technique, which combines GC with human olfaction, provides a viable strategy to distinguish aroma-active compounds (van Ruth 2001; Delahunty et al. 2006). Aroma extract dilution analysis using GC-O can be used to determine the odor active value (OAV) of each VOC, defined as the concentration/odor threshold ratio for assessing its contribution to aroma in a sample (d’Acampora Zellner et al. 2008; Brattoli et al. 2013; Cai et al. 2014). VOCs with high OAVs are strong candidates of key odorants, but we should pay attention that there are some VOCs with low OAVs as important contributors to aroma formation. Previous studies of beer aroma showed the synergistic contributions of multiple odorants including VOCs with low OAVs (sub-threshold components), but not only VOCs with high OAVs, for construction of the well-balanced characteristics of beer aroma (Kishimoto et al. 2018). In the study of Pontianak orange peel oil, VOCs whose OAVs could not be obtained were finally identified as key compounds in its aroma (Dharmawan et al. 2009). These mean that it is not enough to examine the OAV of each VOC for the identification of key odorants in natural products.

Omission test is a powerful technique to determine whether some VOCs are key odorants in complex VOC mixtures (Grosch 2001; Weiss and Vickers 2018). In this procedure, a standard odor of the mixture is prepared by mixing all VOC compositions according to the results of GC/MS-based qualitative and quantitative analyses of VOCs emitted from samples. Omission samples are prepared by mixing VOCs without one or more VOCs to determine their contributions to aroma formation. Then, panelists compare odor qualities between the standard and omission samples by sensory evaluation. If the qualities are different between samples, the omitted VOCs are taken to be key odorants responsible for the characteristic aroma of the VOC mixture. However, this process has some disadvantages. The preparation of omission samples is laborious. In most cases, it is difficult to identify all VOC components of natural products by GC/MS due to the dissimilarities between analytes and compounds registered in GC/MS libraries such as NIST and Wiley. Some VOCs are also unavailable from chemical suppliers, and de novo synthesis of such VOCs can waste resources, costs, and time if they do not contribute to the formation of aroma. Technical skill is necessary for the manual preparation of VOC mixtures with complex chemical compositions.

**Fig. 1.**
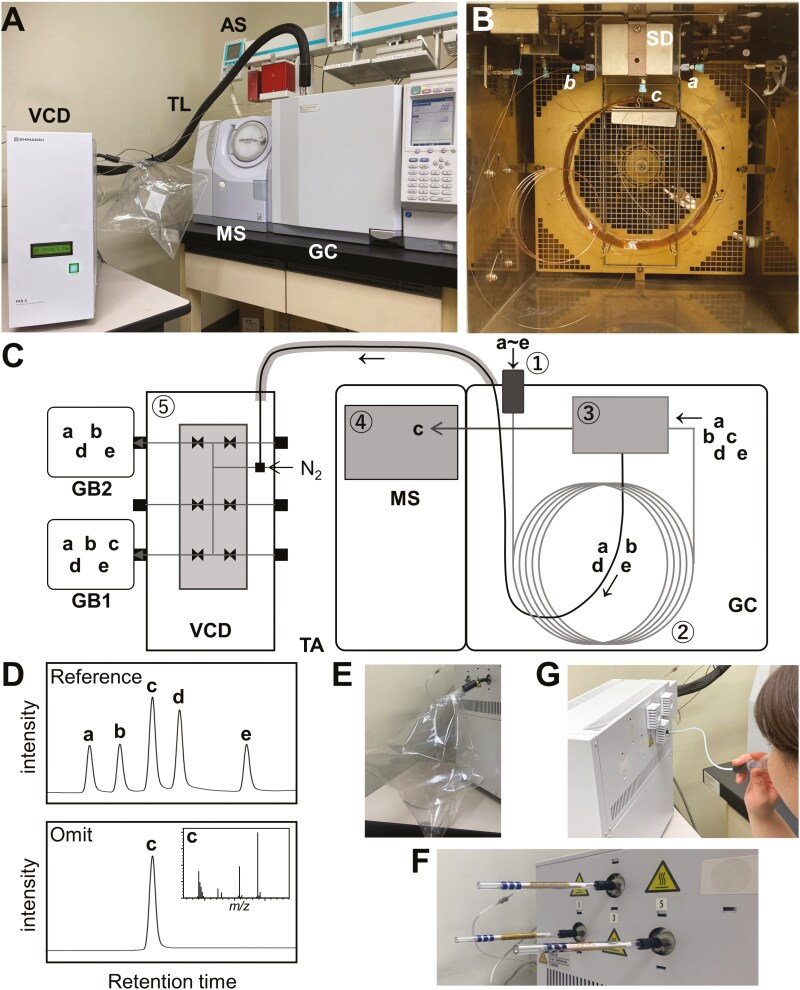
Outline of the developed GC/MS-based VOC collection/omission system. A) The system is composed of a GC autosampler (AS), GC/MS, a heated transfer line (TL), and a VCD. B) A switching device (SD) is installed in GC for change the gas flow routes from the separation column (*a*) to between MS (*b*) and VCD (*c*). C) A schematic image for preparing omission samples and for purifying compounds from 5 volatile organic compounds (a–e) using the system. The first step is to obtain reference TIC in the MS (D, reference) by transferring the 5 compounds to MS. To prepare the reference gas sample for sensory evaluation, all of compounds are collected in a gas bag (GB1) attached to an opened port of the VCD E). To omit compound c from the VOC mixture, it is transferred to the MS and others are collected in GB2. In MS monitoring, compound c is detectable (D, Omit). F) VOCs are collectable in Tenax TA absorbent tubes by attaching them in opened ports of VCD. G) VCD can be also used as a sniffer for sensory evaluation.

To overcome these problems and facilitate omission test, the present study developed a GC/MS-based VOC collection/omission system that can modify chemical compositions in VOC mixtures easily and rapidly without the need to identify chemical structures of components and preparation of authentic compounds. The developed system consists of a GC, a switching unit, MS, and a VOC collection device (VCD) (Fig. 1). The VCD connects to the GC capillary column via a switching unit that also connects to the MS. By switching gas flow routes from MS to the VCD, VOCs separated in the GC are collected into gas bags or absorbent materials attached to the VCD. Omission samples without one or more VOCs can be prepared by transferring only targeted VOCs to the MS and other VOCs to the gas bag or absorbent materials. We first evaluated the resolution and recovery of VOCs in the developed system. Then, we examined the ability of the system to produce omission samples by objectively evaluating using domestic cats as subjects that can detect bioactive compounds from complex odor mixtures of cat-attracting plants. Finally, we tested another application, in which some VOCs of essential oil of lime were replaced by orange VOC components to find key odorants for the lime aroma. The developed system can potentially improve analytical methods for the identification of key odorants in natural products with complex VOC compositions.

## Materials and methods

### Development of a GC/MS-based VOC collection/omission system

This study developed a VOC collection/omission system by improving a GC/MS device (QP-2010 Ultra, Shimadzu Co., Kyoto, Japan) equipped with a CTC Analytics CombiPal autosampler (Zwingen, Switzerland). A switching unit (Shimadzu Co.) was installed in the GC to separate the gas flow route from the separation column (DB-Wax, 30 m × 0.25 mm internal diameter, 0.25 µm film thickness; Agilent Technologies Inc., Santa Clara, CA, USA) to a capillary column (3 m × 0.18 mm internal diameter) connected to the MS and a capillary column (2 m × 0.32 mm internal diameter) connected to the VCD via a 1.5-m transfer line (TL) heated at 250 °C. The VCD had six ports for releasing the separated VOCs from the GC. Each port was located immediately after a pneumatic valve that released the VOCs when opened. As shown in Fig. 1E, G, and F, plastic gas bags (Omi Odor Air Service Co., Ltd., Shiga, Japan), Tenax TA absorbent tubes (Shimadzu Co.), and gas masks (Shimadzu Co.) could be attached to the ports. In this study, the GC/MS was operated using helium as the carrier gas with a column flow of 1.5 ml/min. The GC oven temperature was maintained at 40 °C for 2 min, then increased to 250 °C at a rate of 8 °C/min and held at 250 °C for 6.75 min. The MS was operated in electron impact mode (70 eV) at an ion-source temperature of 200 °C. Mass spectra were obtained in full-scan mode from *m/z* values of 35–500. GC/MS Solution software (ver. 4.53; Shimadzu Co.) was used to operate the switching unit to change the gas flow routes and for total-ion chromatogram (TIC) peak identification.

### Examination of peak resolution in the developed system

**Fig. 2.**
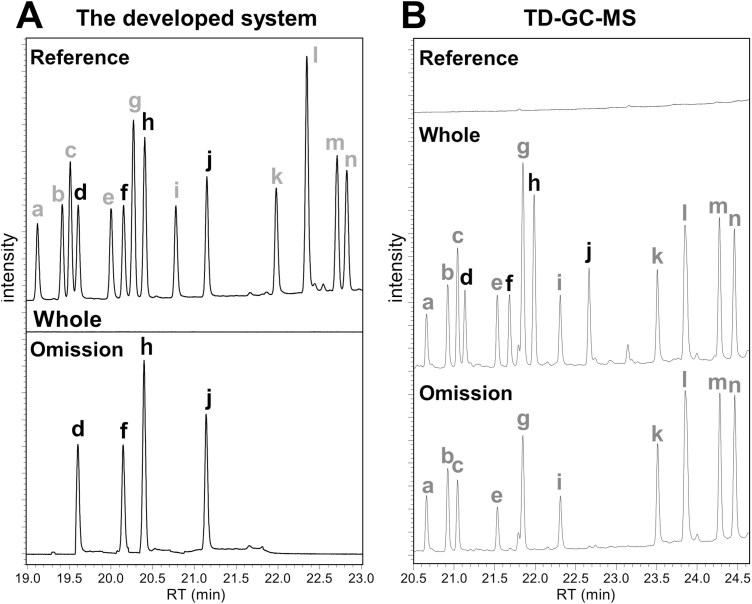
Peak resolution of VOCs in the developed system. A) MS monitoring in the developed system attaching Tenax TA in the opened port of the VCD detected 14 compounds by connecting the separation column to the MS (Reference), no peak by connecting it to the VCD (Whole), and 10 compounds by transferring the other 4 compounds to the MS (Omission). B) TICs of TD-GC/MS analyses of the Tenax TA tubes corresponding to Reference, Whole, and Omit. Peaks are corresponding to following chemicals. a) pentanoic acid, b) (*E*)-citral, c) 2-ethyl butanoic acid, d) 2-methyl pentanoic acid, e) 3-methyl pentanoic acid, f) 4-methyl pentanoic acid, g) *p*-cumic aldehyde, h) perilla aldehyde, i) hexanoic acid, j) 2-methyl hexanoic acid, k) 4-methyl hexanoic acid, l. 2-ethyl hexanoic acid, m) *cis*-jasmone, n) *o*-anisaldehyde.

The peak resolution of the GC/MS-based VOC collection/omission system was examined using the 14 compounds shown in Fig. 2, which were purchased from Tokyo Chemical Industry Co., Ltd. (Tokyo, Japan). First, to obtain reference TICs of the 14 compounds, the autosampler injected one microliter of the mixture (12.5 µM of each compound) into the GC/MS-based VOC collection/omission system that connected the separation column to the MS via the switching unit during the run. Next, to recover all of the 14 compounds using a Tenax TA tube attached to an open port of the VCD, the autosampler injected 1 µl of the mixture into the system that connected the separation column to the device via the switching unit during the run. Finally, to omit four compounds from the mixture, the autosampler injected 1 µl of the mixture into the system that connected the separation column to the VCD whose open port was attached to a Tenax TA tube and changed the gas flow route from the device to the MS at 21.17–22.26 min for 2-methylpentanoic acid, 21.72–21.81 min for 4-methylpentanoic acid, 22.01–22.10 min for perilla aldehyde, and 22.70–22.79 min for 2-methylhexanoic acid.

To check the peak resolution of VOCs in the developed system, the Tenax TA tubes were analyzed in another GC/MS using a thermal desorption device (TD-20; Shimadzu Co.). VOCs trapped in the tubes were desorbed at 250 °C by purging with helium gas at 60 ml/min for 10 min using the TD-20 and were introduced directly into the GC/MS (QP-2010 Ultra device, Shimadzu Co.) by installing a DB-WAX column (60 m × 0.25-mm internal diameter, 0.25-µm film thickness; Agilent Technologies Inc.). All samples were introduced in splitless mode. The oven temperature of the GC was maintained at 40 °C for 2 min, then increased to 250 °C at a rate of 8 °C/min, and held at 250 °C for 6.75 min. The MS was operated as described above. The Shimadzu GC/MS Solution software was used to process the raw data, and provide TIC peak identification, and peak area measurement.

### Examination of recovery of VOCs in the developed system

To examine the VOC recoveries in the GC/MS-based VOC collection/omission system, we tested 87 compounds (see Supplementary Fig. 1). Citronellal, phenyl sulfide, and *trans*-3-hexen-1-ol were purchased from Sigma Aldrich (St. Louis, MO, USA). Butanoic acid and *n*-decane were acquired from Nacalai Tesque, Inc. (Kyoto, Japan). The other compounds were procured from Tokyo Chemical Industry Co., Ltd. (Tokyo, Japan). The 87 compounds were separated into three mixtures to avoid overlapping peaks in the GC/MS TIC. Mixture A was composed of 45 compounds categorized as aldehydes, fatty acids, and other compounds with nitrogen or sulfur; mixture B included 24 compounds classified as alcohols and phenols; and mixture C comprised 18 compounds characterized as alkanes. Each compound was dissolved at 12.5 µM in *n*-hexane in the mixture. The autosampler injected 1 µl of the mixtures into the system that connected the separation column to the VCD with a Tenax TA tube in an opened port during the run. These Tenax TA tubes and another Tenax TA tube spiked with 1 µl of each mixture were analyzed by TD-GC/MS, as described above. The recovery of each VOC was calculated as a percentage as follows: (peak area of the compounds collected in the Tenax TA tube determined by TD-GC/MS)/(peak area of compounds collected in the Tenax TA tube spiked with the same amount of the mixture) × 100%. Recovery was expressed as 100%, if the calculated percentage exceeded 100%.

### Preparation of omission samples from silver vine extract

To test the quality of the omission samples prepared by the developed system, we prepared the samples from organic solvent extracts of silver vine, a plant known for attracting cats, as it emits bioactive iridoids, such as *cis-trans* nepetalactol, isodihydronepetalactone, isoiridomyrmecin, and dihydronepetalactone, that induce the characteristic rubbing and rolling over the plant behavior in cats (Uenoyama et al. 2022). The system omitted the iridoid compounds from the plant extract, and the bioactivity of the sample was examined in behavioral bioassays.

The lipid extract of silver vine was prepared at a concentration of 1 g leaves per milliliter in *n*-hexane, according to the method used in our previous report (Uenoyama et al. 2021). To obtain the reference TIC of VOCs contained in the extract, 4 µl of the silver vine extract was injected into the GC/MS-based VOC collection/omission system that connected the separation column to the MS. Bioactive iridoids such as *cis-trans* nepetalactol, isodihydronepetalactone, isoiridomyrmecin, and dihydronepetalactone were identified by a comparison of the mass spectra and retention time between the extract and the synthesized compounds (Uenoyama et al. 2023). Nine candidate iridoids were identified, based on the high similarity of their mass spectra with iridoids reported in previous studies (Sakai et al. 1980a, 1980b; Lichman et al. 2019, 2020).

To prepare a positive control sample that contained bioactive iridoids to cats, 4 µl of the silver vine extract was introduced into the system that connected the separation column to the VCD equipped with a Tenax TA tube to an opened port. After one injection, the recovery of VOCs was analyzed by TD-GC/MS of the Tenax TA tube. The run was then repeated eight times in the same Tenax TA tube for the following sensory evaluation in cats.

To prepare omission samples containing VOCs other than the 13 iridoids (*cis-trans* nepetalactol, isodihydronepetalactone, isoiridomyrmecin, dihydronepetalactone, and 9 candidate iridoids), 4 µl of the extract was introduced to the system that connected the separation column to the VCD with a Tenax TA tube from 10 min from the GC/MS run. The connection was changed to omit the iridoids from the device to the MS as follows: at 21.68–21.91 min, 22.56–22.95 min, 23.90–24.73 min, 24.97–25.14 min, 25.21–25.36 min, 26.00–26.58 min, 26.67–26.83 min, and 27.49–27.75 min. After checking the quality of the omission samples in the Tenax TA tube using TD-GC/MS, the run was repeated eight times to prepare the sample for the following sensory evaluation in cats. VOCs trapped in each Tenax TA tube were extracted by 2.4 ml of diethyl ether (reagent grade, ≥ 99.5% purity; FUJIFILM Wako Pure Chemical).

### Sensory evaluation in cats

To compare the behavioral response of domestic cats toward the positive control and the omission samples, 6 healthy mixed-breed domestic cats kept at Iwate University participated in the sensory evaluation as subjects. For each subject cat, 2 filter papers (Advantec qualitative no. 1, 70 mm; Toyo Roshi Kaisha Ltd., Tokyo, Japan) were soaked with 400 μl of VOC extracts of the positive control and omission samples. After solvent evaporation, the papers were attached to the bottom of Petri dishes (diameter: 9 cm; AS ONE Corp., Osaka, Japan). Subjects were placed in individual test cages (93 cm × 63 cm × 59 cm; DCM Co., Ltd., Tokyo, Japan) for a few minutes before the sensory evaluation to settle. Petri dishes were fixed on the left and right side (randomized) of the cage floor with gummed cloth tape simultaneously. The behavior of each cat was recorded using a digital video camera (Handycam HDR-CX680; Sony, Tokyo, Japan) placed in front of the cage, and the duration of sniffing of the VOC extracts and that of rubbing and rolling its face, head, and body against them were counted using Behavioral Observation Research Interactive Software (BORIS, version 8.20.3; Friard and Gamba 2016). A Wilcoxon matched-pair signed ranks test (two-tailed, exact *P* value) was used to compare the duration of sniffing and that of rubbing and rolling toward all VOCs versus VOC-eliminated iridoids using SPSS (version 29.0.0.0; IBM Corp., Armonk, NY, USA). The Animal Research Committee of Iwate University approved all sensory evaluation in cats.

### Replacement of partial VOCs between lime and orange essential oils

Finally, the system was used to identify key odorants from lime essential oil. At first, we looked for the lime VOCs whose replacement by the orange VOCs would lack the characteristic lime aroma. Next, based on the results, we prepared omission samples from the lime essential oil. These samples were evaluated by the triangle gas bag test.

To prepare samples for olfactory sensory evaluation, reference TICs of lime and orange essential oils, which were commercial products prepared using the compression method in TREE OF LIFE Co., Ltd. (Tokyo, Japan), were obtained by injection of 2 µl of each sample (×200 dilution in *n*-hexane) into the GC/MS-based VOC collection/omission system, as described above. To obtain whole aroma samples of lime (LLLL) and orange (OOOO), that is, lime and orange essential oils, respectively, were introduced to the system that transferred VOCs from the separation column to the VCD with Tenax TA tubes or gas bags. The Tenax TA tubes and gas bags were used for quality checks by TD-GC/MS and olfactory sensory evaluation, respectively. To replace some of the lime VOCs with orange VOCs, for example, OLLL shown in Fig. 5B, essential oil of lime was first introduced into the system. The lime VOCs eluted before 12.3 min were transferred to the MS. By switching the gas flow route from the MS to the VCD after 12.3 min, other eluted VOCs were collected in the Tenax TA tube or a bag. Next, the essential oil of orange was injected into the system. The VOCs eluted before 12.3 min were collected in the same Tenax TA tube or the bag containing partial lime VOCs. Other VOCs eluted after 12.3 min were transferred to the MS. In the same manner, samples OOLL and OOOL were prepared by replacing VOCs between the 2 oils by switching the gas flow route. Compounds in lime and orange essential oils were tentatively identified by searching of the Wiley MS library (seventh edition) and the NIST library (eighth edition).

### Preparation of omission samples from lime essential oil

To prepare omission samples of the lime essential oil, 2 µl of the oil (×200 dilution in *n*-hexane) was introduced to the system, in which the switching unit changed the gas flow route as described in the following: Omit 1: to the VCD at 5.00–12.30 min and at 15.00–35.00 min and to the MS at 0–5 min and at 12.30–15.00 min; Omit 2: to the device at 5.00–15.00 min and 20.00–35.00 min and to the MS at 0–5.00 min and at 15.00–20.00 min; Omit 3: to the device at 5.00–16.70 min, at 16.95–17.24 min, at 17.37–17.65 min, at 17.78–18.69 min, and at 18.90–35.00 min and to the MS at 0–5.00 min, at 16.70–16.95 min, at 17.24–17.37 min, at 17.65–17.78 min, and at 18.69–18.90 min; and Omit 4: to the device at 5.00–15.00 min, at 16.70–16.95 min, at 17.24–17.37 min, at 17.65–17.78 min, at 18.69–18.90 min, and at 20.00–35.00 min and to the MS at 0–5.00 min, at 15.00–16.70 min, at 16.95–17.24 min, at 17.37–17.65 min, at 17.78–18.69 min, and at 18.90–20.00 min.

### Olfactory sensory evaluation in human subjects

In the first trial, mixtures of lime and orange VOCs were evaluated by nine panelists (6 males and 3 females, aged between 20 and 48 years) who were recruited from the Iwate University campus. The aroma qualities and characteristics in the reference aromas of lime and orange essential oils were decided by discussion among the panelists. Then, the panelists sniffed the gas bags containing mixtures of lime and orange aroma prepared by the GC/MS-based VOC collection/omission system, and evaluated which samples smelled like lime or orange aroma by comparing their sample with the reference aroma. Then, the panelists discussed aroma profiles of the samples and decided the VOC fractions necessary to produce the lime aroma for further omission tests.

In the second and third trials, the 9 panelists evaluated omission samples prepared from lime essential oil using the developed system as described above by triangle odor gas methods. They were presented with 3 gas bags consisting of 2 referenced samples and 1 omission sample and performed an olfactory evaluation to determine which one was an omission sample. The data of these triangle tests were statistically analyzed using the one-tailed binomial test (probability: 0.333) (Sinkinson 2017). This experiment was conducted according to the Declaration of Helsinki for Research involving Human Subjects and received approval from the Ethics Committee of Iwate University. The panelists gave verbal informed consent by M.M.

## Results

### Outline of the GC/MS-based VOC collection/omission system

The developed system is composed of a GC autosampler, GC with a switching unit, MS for chemical analysis, a heated TL, and a VCD (Fig. 1). After injecting samples into the GC using the autosampler, a capillary column separates VOCs from the sample. Then, a switching unit located at the end of the column changes the gas flow routes between MS and the VCD in a manner dependent on retention time. MS monitoring enables confirmation that VOCs whose peaks have disappeared in TIC are transferred to the VCD. The VCD can introduce air or nitrogen at a flow rate of 30–100 ml/min from the metal tubes connecting the device to the end of the capillary column, which enables us to transfer VOCs eluted at a flow rate of 1–3 ml/min from the capillary column (0.25–0.32 mm diameter) to the gas bag or absorbent material quickly. In the VCD, 6 pneumatic valves are located immediately before the 6 ports that emit VOCs to the atmosphere. VOCs are eluted from the opened ports of the pneumatic valves and collected in the gas bag (Fig. 1E) or absorbent like Tenax TA (Fig. 1F) attached to the ports, where separated VOCs by GC are again mixed with other VOCs. As the VCD requires a port to vent the carrier gas when it does not collect VOCs, we use 5 ports for sample preparation. In addition, by attaching gas masks to 2 opened ports, 2 panelists could smell each separate VOC and evaluate it (Fig. 1G).

### Peak resolution of VOCs in the developed system

The autosampler injected a mixture of 14 VOCs into the developed system with absorbent tubes containing Tenax TA attached to an opened port in the VCD. Once all VOCs were transferred to the MS via the switching unit, 14 peaks were detected as references in TIC (Fig. 2A). In analysis of the Tenax TA tube using another thermal desorption (TD)-GC/MS, TIC had no peak (Fig. 2B, reference), confirming that few VOCs were transferred to the VCD. In the second injection, as all 14 VOCs had been transferred to the VCD, no peak was detected in MS monitoring (Fig. 2A, whole). By contrast, subsequent TD-GC/MS analysis of the Tenax TA tube detected the 14 VOCs in a similar pattern to the reference TIC (Fig. 2B, whole). With the third injection, the system omitted 4 of 14 VOCs from the mixture. During sample preparation, MS monitoring detected only the 4 VOCs in TIC. TD-GC/MS of the Tenax TA tube that collected the 11 VOCs detected in TIC. Importantly, not only the single peak without other peaks within 0.2 s of the retention time (j) but also peaks with other peaks within 0.1 s of the retention time (d, f, and h) were completely omitted in the developed system.

### Recovery of 87 VOCs in the developed system

**Fig. 3.**
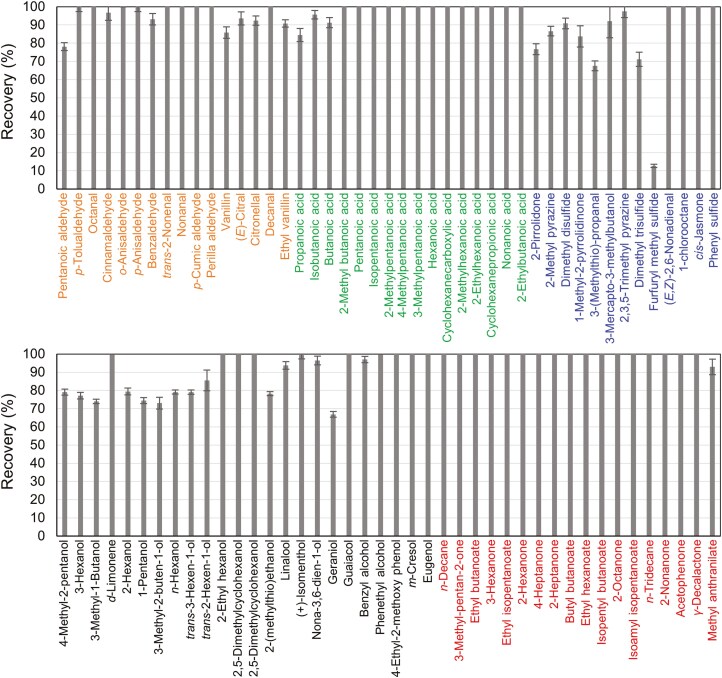
Peak recoveries of 87 VOCs in the developed system. Bar graphs show recoveries (mean ± S.E.%) of 87 VOCs in the developed system. Recovery was expressed as 100% if the calculated percentage exceeded 100%. Orange, green, blue, black, and red indicate groups of aldehydes, fatty acids, alcohols/phenols, alkanes, and other compounds with nitrogen or sulfur, respectively. TICs of the VOCs are available in Supplementary Fig. 1.

Figure 3 shows the recoveries (%) of 87 VOCs. The average total recovery of the 87 VOCs was 93%. The recoveries in aldehydes, fatty acids, alcohols/phenols, and alkanes were 95.7 ± 0.4%, 98.2 ± 0.3%, 88.4 ± 0.5%, and 99.6 ± 0.1%, respectively. Recovery was lowest for nitrogen- or sulfur-containing VOCs (83.0 ± 1.8%) as compared to other VOCs. Especially, recoveries of some sulfur-containing VOCs expressed low values, such as 67.5 ± 2.7% in 3-(methylthio)-propanal, 71.1 ± 3.9% in dimethyl trisulfide, and 12.7 ± 0.9% in furfuryl methyl sulfide.

### Evaluation of quality of omission samples by domestic cats

To evaluate whether the developed system can prepare high-quality samples for omission tests, the prepared samples were objectively evaluated by domestic cats, whose olfactory system is superior to that of humans (Linda 2003). Briefly, a *n*-hexane extract of cat-attracting silver vine plant was introduced into the developed system to prepare VOC samples with or without plant iridoids, and then both samples were presented simultaneously to the cats to examine their behavioral responsiveness to the samples. As iridoids, such as *cis-trans* nepetalactol, isoiridomyrmecin, dihydronepetalactone, and isodihydronepetalactone, are bioactive compounds known to induce characteristic rubbing and rolling over behaviors in cats (Uenoyama et al. 2021, 2022), we expected that cats would exhibit the characteristic response to the sample with iridoids rather than that without iridoids.

**Fig. 4.**
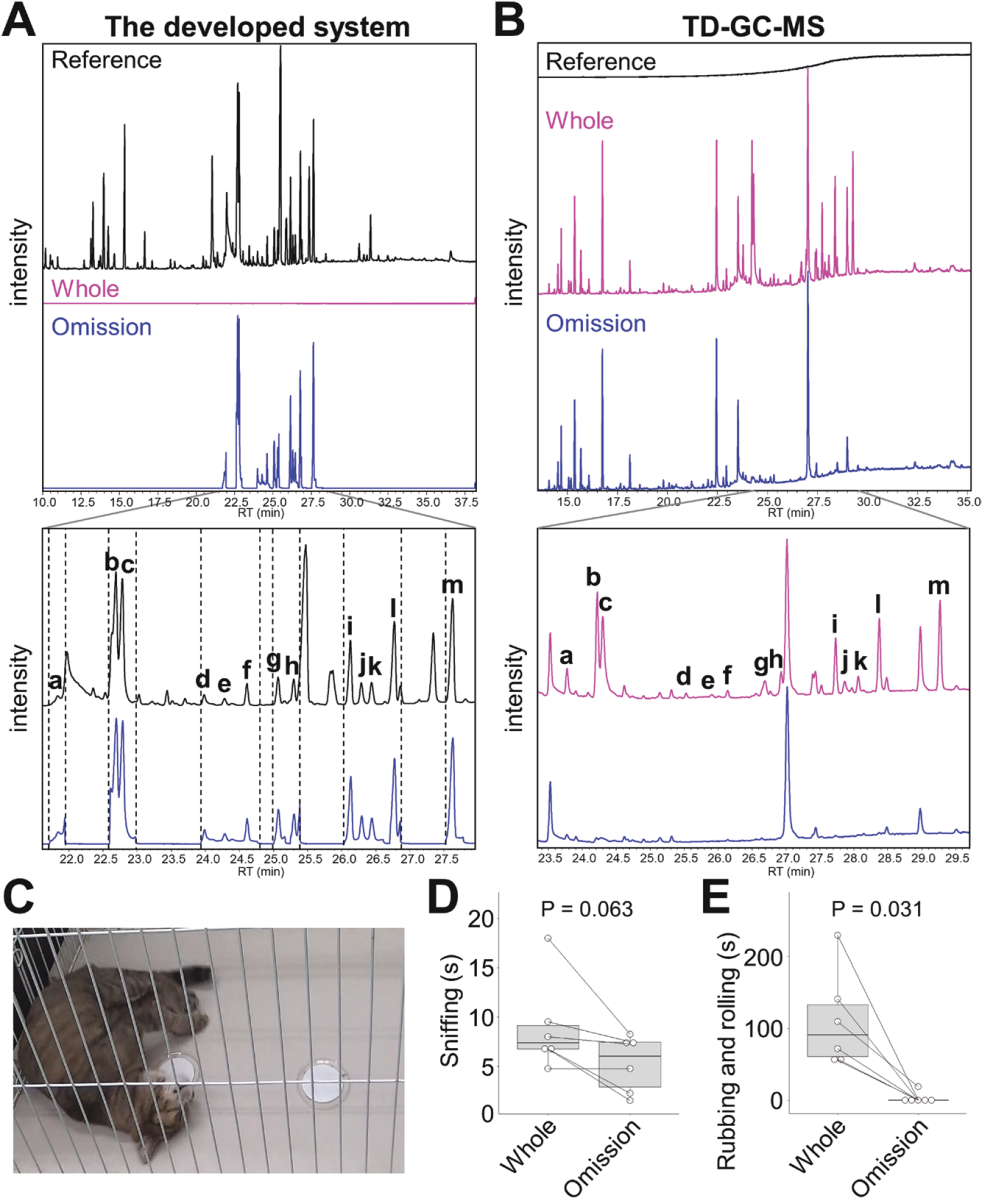
Objective evaluation of omission samples prepared in the developed system by cats. A) Upper box: MS monitoring in the developed system attaching Tenax TA tubes in the opened ports obtained TICs by transferring all VOCs of the silver vine extracts to the MS (Reference) or to the VCD (Whole) or the 13 iridoids and the other VOCs to the MS and to the VCD, respectively (Omission). Lower box: Enlarge TICs of Reference and Omission. B) Upper box: TICs of TD-GC/MS analyses of the Tenax TA tubes corresponding to Reference, Whole, and Omission. (A and B) Lower box: Enlarged TICs of Whole and Omission. A and B) b, and c. candidates of *trans-trans* iridodial, f. *cis-trans* nepetalactol, h. isodihydronepetalactone, i. isoiridomyrmecin, k. dihydronepetalactone, l. candidate of isoepiiridomyrmecin, m. isoneonepetalactone, others. unknown candidate iridoids. C) Sensory evaluation in domestic cats presenting toward papers impregnated with all VOCs of silver vine extract and the VOCs eliminated the 13 iridoids. D and E) Duration of sniffing D) and rubbing and rolling E) toward Whole and Omission in 6 cats. *P* values from Wilcoxon matched-pair test, two-tailed. See also Supplementary Fig. 2.

First, silver vine leaf extract was injected into the system in which all VOCs were introduced into the MS for TIC as a reference (Fig. 4A). Next, all VOCs of the extract were collected in the Tenax TA tube attached to the opened port of the VCD, which was confirmed by the lack of peaks in TIC in MS monitoring. To prepare the omission sample, four iridoids (*cis-trans* nepetalactol, isodihydronepetalactone, isoiridomyrmecin, and dihydronepetalactone) and 9 other candidate iridoids, including *trans-trans* iridodial, isoepiiridomyrmecin, and isoneonepetalactone, found according to the MS spectra (Sakai et al. 1980a, 1980b; Lichman et al. 2019, 2020) were transferred into the MS and the other VOCs were collected in the Tenax TA tube attached to the opened port of the VCD (Supplementary Fig. 1). TD-GC/MS analyses of both tubes confirmed that VOCs with and without the 13 iridoids were successfully prepared (Fig. 4B). VOCs trapped in the Tenax TA tubes were extracted using diethyl ether, and then papers soaked with these extracts were presented simultaneously to the cats as shown in Fig. 4C. Although there were no significant differences in sniffing duration between VOC-soaked papers with and without iridoids (Fig. 4D; exact *P* = 0.063), with the exception of one cat, the animals exhibited the characteristic rubbing and rolling over behavior toward VOCs with iridoids but not to those without them (Fig. 4E; exact *P* = 0.031). These results indicated that few iridoids were contained in the omission sample of silver vine leaf extract.

### Identification of key aroma compounds from lime essential oil

Essential oils of citrus fruits are among the most commonly used aromatic oils in commercial products, such as beverages, foods, and perfumes. Most citrus fruits emit limonene as a major component, which contributes to the unique “citrus aroma” rarely found in other fruit species (Vieira et al. 2018). It is easy to distinguish aromas among citrus fruits, for example, between lime and orange, by olfaction, indicating that compositions of VOCs other than limonene characterize the specific aromas of each citrus fruit. In preliminary studies, all VOCs collected from lime and orange essential oils using the developed system were sensed as lime aroma and orange aroma, respectively. We hypothesized that lime aroma could be changed gradually to orange aroma by replacing lime-based VOCs with orange VOCs. To identify key odorants responsible for the characteristic lime aroma, in the first step, we attempted to find the lime VOCs whose replacement by orange VOCs would result in the change in specific aroma.

**Fig. 5.**
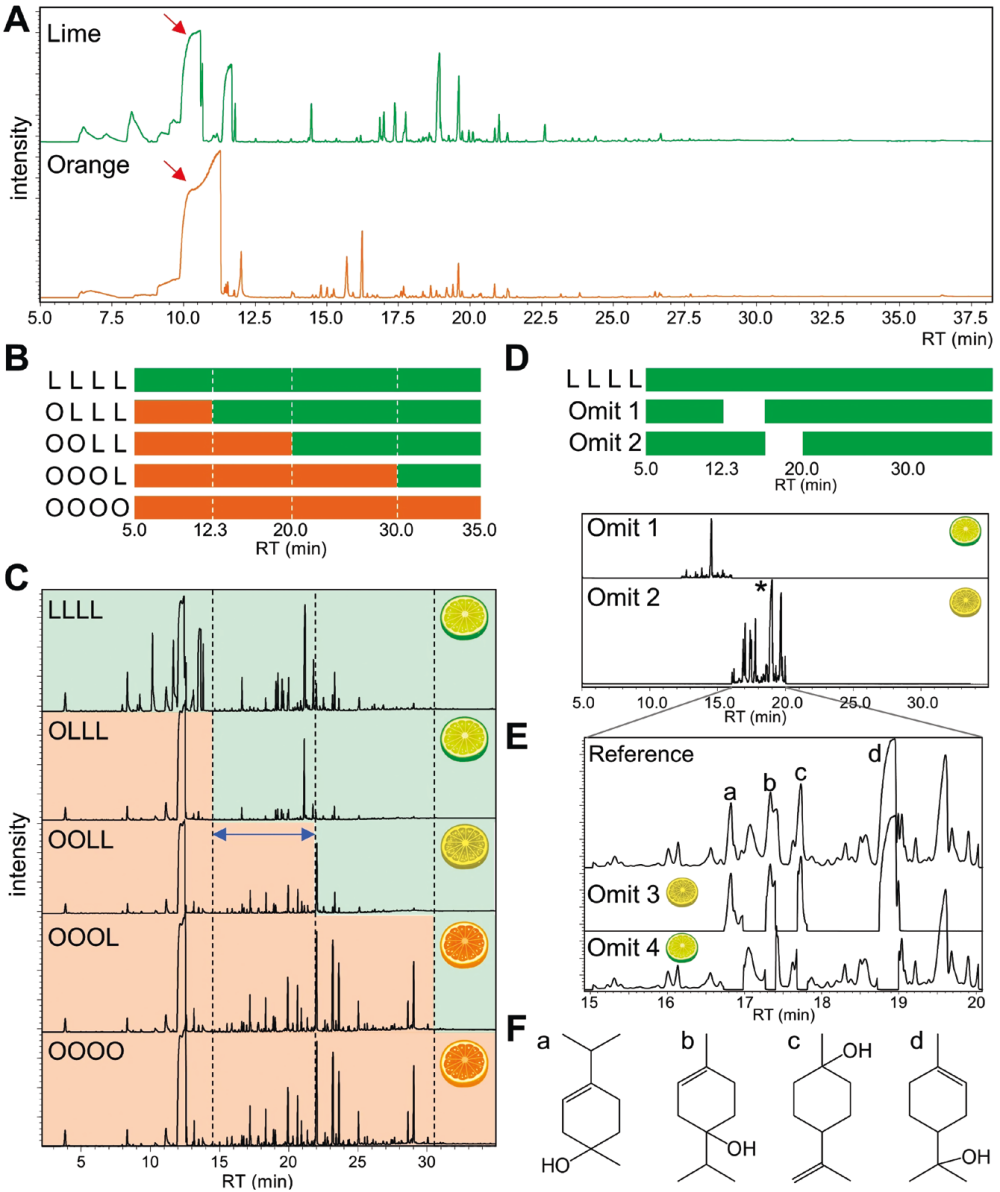
Identification of key aroma compounds of lime using the developed system. A) Reference TICs of the lime and orange essential oils. B) Pattern diagrams of 5 samples obtained from lime and orange essential oils in the developed system. LLLL and OOOO were whole samples containing only lime and orange VOCs, respectively. In OLLL, OOLL, and OOOL, lime VOCs eluted before 12.3 min, 20.0 min, and 30.0 min, respectively, were replaced by orange VOCs eluted at the same retention time. C) TICs of TD-GC/MS analyses of Tenax TA tubes corresponding to LLLL, OLLL, OOLL, OOOL, and OOOO. D) Upper: Pattern diagram of LLLL and 2 omission samples (Omit 1 and 2). Lime VOCs eluted at 12.3–15.0 min and at 15.0–20.0 min were omitted by transferring into the MS in Omit 1 and Omit 2, respectively, and the rest of VOCs were transferred into the VCD. Lower: TICs of Omit 1 and 2 obtained in the GC/MS-VOC collection system. E) Enlarged TICs of the lime essential oil obtained in GC/MS-VCD, in which all VOCs were transferred to the MS (Reference) or 4 terpineols (a. 1-terpineol, b. 4-terpineol, c. *β*-terpineol, d. *α*-terpineol) (Omit 3) or lime VOCs eluted between 15 min and 20 min (excluding the 4 terpineols) (Omit 4) were omitted. F) Chemical structures of the 4 terpineols. Characters are corresponding to compounds shown in (E).

Figure 5A shows the TICs of lime and orange essential oils. Limonene formed a major peak for both samples (arrows). According to the reference TICs, we prepared 5 VOC mixtures derived from lime and orange essential oils using the developed system (Fig. 5B). Two samples consisted of only lime (LLLL) or orange VOCs (OOOO). Three samples, OLLL, OOLL, and OOOL, were mixtures of lime and orange VOCs. Figure 5C shows the TICs of TD-GC/MS of Tenax TA tubes of the 5 samples. The TICs of OLLL, OOLL, and OOOL were the same as the reference TIC of the orange sample (OOOO) until 15, 22, and 30.5 min, respectively. The remaining TICs were matched with LLLL, confirming the qualities of samples in which partial lime VOCs were replaced with orange VOCs (Fig. 5C). In olfactory evaluations, they sensed LLLL-like and OOOO-like aromas in samples OLLL and OOOL, respectively. In contrast, all 9 panelists classified the odor quality of sample OOLL as an unknown citrus aroma that was neither LLLL nor OOOO aroma, suggesting that VOCs eluted between 12.3 and 20 min in the system included key odorants characterizing lime aroma.

To identify the key odorants of lime aroma, we further prepared omission samples in which some of the volatile compounds eluted between 12.3 and 20 min were omitted from the lime volatile compounds by the developed system and evaluated as to whether the 9 panelists could distinguish between the omission samples and LLLL using triangle odor bag methods. By injecting lime essential oil into the system, all VOCs were sampled in a Tenax TA tube or a gas bag that was attached to the VCD. Two samples, Omit 1 and Omit 2, in which VOCs eluted between 12.3 and 15 min and between 15 and 20 min were omitted, respectively, were also prepared by switching the flow route. In MS monitoring, only omitted VOCs, but not other VOCs, were detectable (Fig. 5D), confirming the qualities of Omit 1 and Omit 2. In the triangle odor bag method using 2 gas bags with LLLL (Reference) and a gas bag with Omit 1, only 4 of the 9 panelists could distinguish LLLL and Omit 1 correctly, indicating that no significant difference in odor qualities was observed between LLLL and Omit 1 (*P* = 0.349). In contrast, the method using the 2 bags with LLLL and a bag with Omit 2, 8 of the 9 panelists could distinguish LLLL and Omit 2, indicating that the odor qualities were significantly different (*P* = 0.001) between LLLL and Omit 2. They expressed the odor quality of Omit 2 as an unknown citrus aroma that was not lime aroma. These results strongly suggest that VOCs eluted between 15 and 20 min in the system included key odorants characterizing lime aroma.

As GC/MS found that Omit 2 lacked some VOCs containing isomers of terpineol, we prepared 2 omission samples, Omit 3 and Omit 4, lacking only isomers of terpineol and VOCs other than terpineols eluted between 15 and 20 min, respectively (Fig. 5E). In the triangle tests using 2 gas bags containing LLLL and 1 gas bag containing Omit 3, 7 of the 9 panelists could distinguish between LLLL and Omit 3 correctly, indicating that the odor qualities were significantly different (*P* = 0.008) between LLLL and Omit 3. In contrast, the test using 2 LLLL bags and a bag containing Omit 4, only 4 of the 9 panelists could distinguish between LLLL and Omit 4 correctly, indicating that no significant difference in odor qualities was observed between the 2 (*P* = 0.349). Based on these results, we concluded that terpineols are the main contributors to lime aroma.

## Discussion

The present study showed the usefulness of the developed GC/MS-based VOC collection/omission system, which is composed of a GC/MS and a switching unit for changing gas flow routes between MS and the VCD. This system can arrange the chemical compositions of VOCs easily and rapidly. In this study, we applied the developed system to produce samples for omission tests and for the replacement of VOC compositions between 2 samples.

We propose the importance of the evaluation of mixtures of VOCs after omitting only one or more VOC constituents rather than individual VOCs using GC-O. In contrast, current aroma research focuses on the improvement of peak separation in GC and sensitivities and MS resolution for comprehensive analysis of all VOC constituents of natural products. Two-dimensional GC/MS (GC × GC/MS) with two columns of different polarity is a powerful tool for VOC profiling of natural products and is capable of detecting hundreds more VOCs than conventional GC/MS (Suzuki et al. 2019; Baldovini and Chaintreau 2020). As there are many VOCs that do not contribute to the characteristic aroma of natural products in mixtures of other VOCs, regardless of efforts to improve techniques for separation of VOCs using GC/MS and GC × GC/MS, it is not always possible to identify key odorants of natural products. In addition, an aroma produced by combining many VOCs is not similar to the original aroma of individual constituents via odorant synergistic effects. For example, in the analysis of marine oil using a GC-trapping-olfactometry approach, a combination of heptanal and (*E*,*Z*)-3,5-octadien-2-one produced a fishy odor, as indicated by a previous study (Marsili and Laskonis 2014). A mixture of vanillin (sweet odor) and isopentanoic acid (unpleasant sock odor) resulted in a pleasant aroma, similar to that of chocolate (Hirasawa et al. 2019). Therefore, we think that the concept of analyzing VOC mixtures by omission, without evaluating the odor of each constitute, is important for aroma research. The use of electric noses with sensors represents one method to evaluate the aromas of natural products without separating each VOC (Zhang et al. 2022); however, it is impossible to identify the chemical structures of key odorants via such analyses. Our developed system enables us to evaluate the odor quality of VOC mixtures with and without individual VOCs and analyze the chemical structures of VOCs by MS. This approach is useful for identifying key odorants from natural products with VOC mixtures and examining which combinations of the contents have synergistic effects in the aroma.

There are lots of studies that carry out omission tests in conventional manners to determine whether one or more VOCs contribute to the aroma formation of natural products (Dharmawan et al. 2009; Steinhaus et al. 2009; Mayr et al. 2014; Lorjaroenphon and Cadwallader 2015; Ma et al. 2017; Sun et al. 2018; Wang et al. 2020, 2023; Chen et al. 2022). For omission test in these studies, samples for olfactory sensory evaluation had been prepared according to data obtained by GC/MS-based qualitative and quantitative analyses and GC-O of natural products. The original aroma of natural products would not be reconstructed by mixing VOCs according to such data as in most cases it is difficult to identify all constituents present in natural products. To overcome these problems, perfumers estimate the missing VOC components and try to reproduce the original aroma by adding them to inadequate mixtures, and also prepare omission samples by removing one or more VOCs in the mixture. Therefore, typical omission test requires expert knowledge and skill obtained by experience as well as a long time for sample preparation. By contrast, the developed system enables nonexperts to prepare samples for omission test with neither identification nor quantification of VOCs or preparation of authentic compounds for reconstruction of samples. Although there are systems that can collect VOCs only to absorbents using a GC fraction collector (Koga et al. 2021), no systems capable of preparing gas samples for olfactory evaluation directly, such as the developed system, have previously been reported.

Peak resolution and recovery of VOCs in the developed system exhibit sufficient performance to prepare gas samples for omission test. Although the recoveries of some VOCs were around 70% to 80%, as olfactory thresholds vary in the logarithmic range in humans (Venstrom and Amoore 1968; Stevens et al. 1988), such recoveries would not pose problems for olfactory evaluations. Unfortunately, recovery of sulfur-containing VOCs, particularly furfuryl methyl sulfide, was not good compared to other VOCs. Although we could not resolve this low recovery in this study, as sulfinert®-treated stainless steel tubes (Hadj Nacer et al. 2014) were used for developing the VCD, it is possible that this loss of detection occurred in the switching unit, pneumatic valves, or ports that are immediately cooled by outdoor air.

GC/MS of essential oils prepared from natural products by steam distillation and TD-GC/MS of Tenax TA concentrating VOCs emitted from them are often carried out to identify key odorants of natural products and foods (Wilkes et al. 2000; Dugo et al. 2005). It is important in GC/MS analysis to confirm that all VOCs introduced into MS can reproduce the original aroma of the products using the developed system. Some VOCs may be produced from other compounds during GC separation and thermal desorption from the absorbents at high temperatures (approximately 200–250 °C) (Arora et al. 1995; Feng et al. 2017) and may pass through the absorbents during collection (Dettmer and Engewald 2002). If the original aroma is not reconstructed in the gas correctly, it may be difficult to detect key odorants in MS due to problems of heat denaturation of VOCs and headspace sampling, and some VOCs needed for reconstruction of the odor would be missed. In this analysis, we found no such problems after introducing silver vine extract and essential oils of lime and orange using the developed system. Making use of the behavioral responsiveness of cats toward gas samples collecting all VOCs contained in silver vine extract, we confirmed that bioactive iridoids were collected in the gas bag in the developed system. Furthermore, we experienced no problems in reproducing the original aroma by introducing essential oils of lime and orange into the system. The developed system therefore also appears to be useful for pre-investigation whether VOCs are thermally decomposed or lost in GC and sample preparation steps before chemical analyses using MS.

Our system will aid research into determining whether characteristic aroma and bioactivity can be explained by only the identified compounds in samples, a common question in studies seeking bioactive compounds from natural products. This study examined whether the developed system can omit iridoids that are bioactive toward cats from the VOC mixture of silver vine extract (Uenoyama et al. 2021, 2022). The advantage of using cats is that they have highly developed olfactory systems, allowing for results that can be considered objectively and statistically (Linda 2003). In preliminary studies, we omitted the well-known bioactive compounds *cis-trans* nepetalactol, isoiridomyrmecin, dihydronepetalactone, and isodihydronepetalactone from VOCs of the extracts but cats showed the response not only toward the VOC mixtures with these compounds but also the omission samples (data not shown). There may be other bioactive VOCs in these omission samples. The bioactive compounds responsible for the characteristic response in cats are iridoids with a 5-membered ring fused to a 6-membered ring with oxygen, which exist as many stereoisomers (Bol et al. 2022). Therefore, we carefully checked the peaks in TIC and found a further nine candidate iridoids including *trans-trans* iridodial, isoepiiridomyrmecin, and isoneonepetalactone in addition to the 4 known iridoids. These bioactivities are not well-known in previous papers (Bol et al. 2022). Finally, we found that the omission sample without the 4 iridoids and the 9 candidate iridoids showed little bioactivity toward cats, although it induced sniffing, strongly suggesting that silver vine extract contains more than 10 iridoids that are bioactive in cats. These observations indicate that the developed system would be useful for identifying bioactive compounds that we have missed in natural products.

In addition to omission test, the developed system can produce artificial aromas that do not exist in natural products by changing VOC compositions between samples. This will also help to screen for key odorants that contribute to distinguishing natural products with similar odors. In this study, we selected essential oils of lime and orange as samples, because they have common citrus aromas but can be easily distinguished by smell, and several studies have reported the VOCs of these fruits. Our experiments showed that lime aroma could be changed to an unknown citrus aroma by replacing lime VOCs eluted between 12.3 min and 20 min in GC with orange VOCs eluted with the same timing. By contrast, replacing major VOC contents of lime, such as *α*-pinene, sabinene, and *β*-pinene, with other orange VOCs had little effect on lime aroma. Finally, omission test revealed that terpineols are important for the characteristic lime aroma in the essential oil of lime. Previous studies have reported that monoterpene derivatives, such as *α*-fenchol, borneol, isoborneol, and *β*-terpineol, have musty, camphor-like odors and together with *α*-terpineol and carvone are considered as off-odors in citrus products, which do not have large OAVs, but contribute to the aroma of distilled lime oil (Chisholm et al. 2003). Our findings support these previous studies. VOCs are classified as top, middle, and base notes according to their odorous characteristics, diffusion rate in air, and volatility (Sharmeen et al. 2021). The top notes are those that are the most volatile and the first perceptible odors (Sharmeen et al. 2021). Our findings indicate that middle notes eluted between 12.3 min and 20 min are important for the characteristic lime aroma rather than top notes that characterize the common citrus aroma. Conventional GC-O can be used to screen for candidate key odorants based on OAV in each VOC but not to determine whether such odorants are major contributors in VOC mixtures. Therefore, our developed system will be useful for improving aroma research in natural products.

We also propose that the developed system is a powerful tool for identifying bioactive compounds, such as pheromones and scent signals in studies of animal olfactory communication. In most previous studies, it was challenging to observe the bioactivity in high-purity compounds isolated from secretions and excretions in physiological amounts (Vogt 2003; Baum 2012). This would suggest that the secretions and excretions contain other ingredients that have synergistic effects for the bioactive compounds but do not possess the necessary bioactivity by themselves (Nojima et al. 2007). Bioactivities of high-purity compounds are usually assessed at non-physiologically high concentrations, which may make the studies questionable (Wyatt 2015). Although the reconstruction of complex odor mixtures by mixing authentic compounds is difficult, the developed system can prepare positive control samples with bioactivity that contain all VOCs emitted from a crude sample; test samples can then be prepared by omitting candidate bioactive compounds from all VOCs. This means that we can test the bioactivity of the candidates at a reasonable physiological amount, which helps to ensure the reliability of the findings. Moreover, the system has the ability to identify multiple bioactive compounds that work in a blend of a specific ratio more easily, by omitting these peaks from all VOCs as compared to traditional purification techniques such as liquid chromatography and preparative GC (Linn et al. 1986; Mori 2010; Yew and Chung 2015).

One limitation of the developed VOC collection/omission system is that it may not be valid for compounds present in complex matrices (like foods), where physical interactions (partitioning) become important. However, it is a powerful tool for compounds in the gas phase in its ability to identify key odorants and bioactive compounds from natural products that emit many VOCs, as well as designed artificial aromas that do not exist in nature. In conclusion, our system will be useful for future aroma research to identify key VOCs from natural products with complex mixtures of VOCs.

## Supplementary Material

bjae007_suppl_Supplementary_Figures_S1-S2

## Data Availability

The data supporting the results of this article will be shared on reasonable request to the corresponding author.
