# Supplementary material for: Development of the gas chromatography/mass spectrometry-based aroma designer capable of modifying volatile chemical compositions in complex odors
Source: Chem Senses. 2024 Feb 22;49:bjae007. doi: 10.1093/chemse/bjae007 (PMC11812034; doi:10.1093/chemse/bjae007)
Supplement: bjae007_suppl_Supplementary_Figures_S1-S2 [file bjae007_suppl_supplementary_figures_s1-s2.pdf]

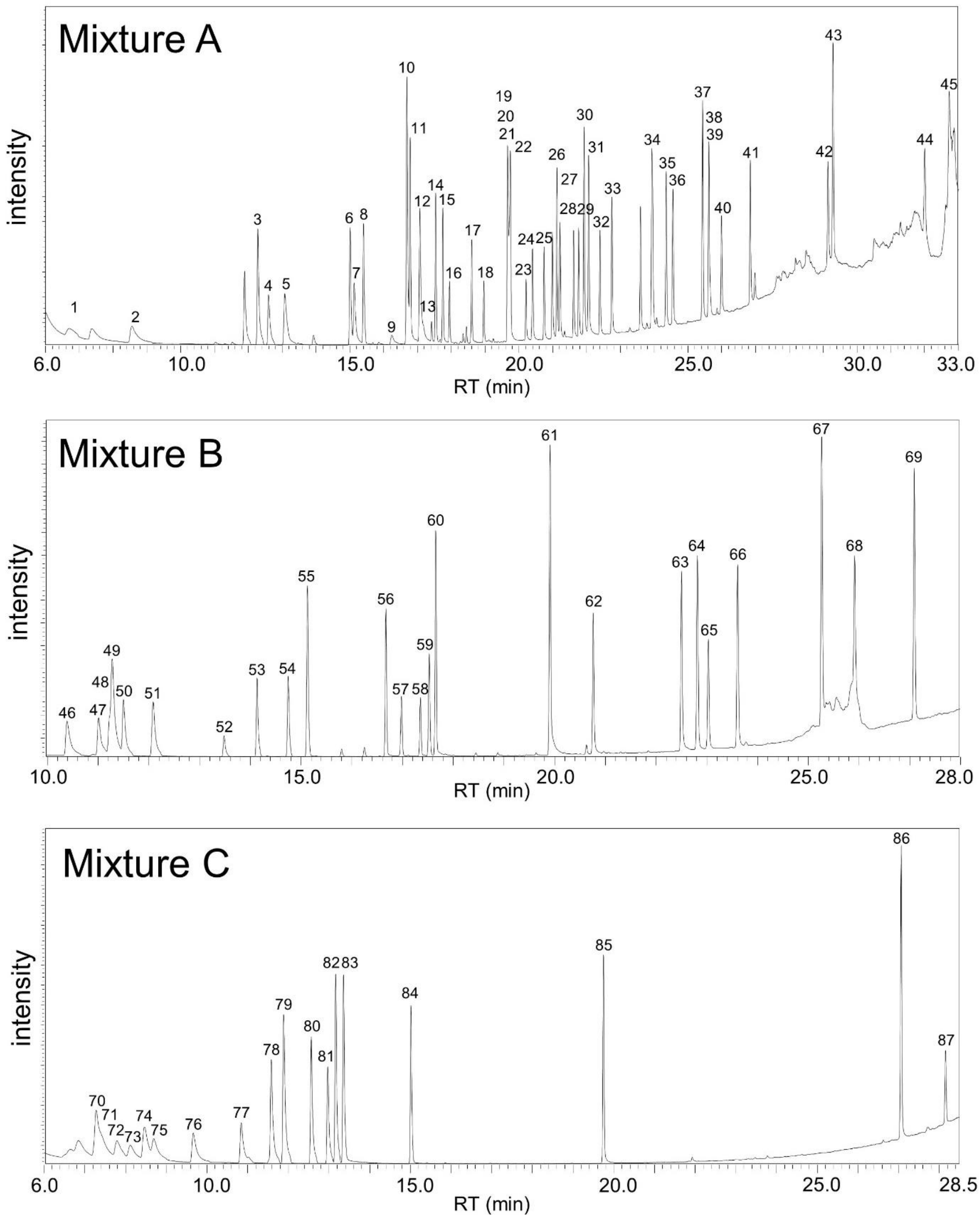

Supplementary Fig. 1. GC-MS total ion chromatograms of 87 VOCs, which were obtained in the GC-MS-based VOC collection/omission system, related to Fig. 3.

Mixture A contained 45 VOCs categorized as aldehydes, fatty acids, and other compounds with nitrogen or sulfur. Mixture B contained 24 VOCs categorized as alcohols and phenols. Mixture C contained 18 VOCs categorized as alkanes. Peak numbers are corresponding to following chemicals. 1. pentanoic aldehyde, 2. dimethyl disulfide, 3. 1-chloro octane, 4. 2-methyl pyrazine 5. octanal, 6. dimethyl trisulfide, 7. nonanal, 8. 2,3,5-trimethyl pyrazine, 9. 3-methylthio propanal, 10. citronellal, 11. furfuryl methyl sulfide, 12. decanal, 13. propanoic acid, 14. benzaldehyde, 15. *trans*-2-nonenal, 16. isobutanoic acid, 17. (*E*,*Z*)-2,6-nonadienal, 18. butanoic acid, 19. isopentanoic acid, 20. 2-methylbutanoic acid 21. 3-mercapto-3-methylbutanol, 22. *p*-tolualdehyde, 23. (*E*)-citral, 24. 1-methyl-2-pyrrolidinone 25. pentanoic

acid, 26. 2-ethylbutanoic acid, 27. 2-methylpentanoic acid, 28. 3-methylpentanoic acid, 29. 4-methylpentanoic acid, 30. *p*-cumic aldehyde, 31. perilla aldehyde, 32. hexanoic acid, 33. 2-methylhexanoic acid, 34. 2-ethylhexanoic acid, 35. *cis*-jasmone, 36. *o*-anisaldehyde, 37. *p*-anisaldehyde, 38. cinnamaldehyde, 39. 2-pyrrolidone, 40. cyclohexanecarboxylic acid, 41. nonanoic acid, 42. cyclohexanepropionic acid, 43. phenyl sulfide, 44. ethyl vanillin, 45. vanillin. 46. 4-methyl-2-pentanol, 47. 3-hexanol, 48. 3-methyl-1-butanol, 49. *d*-Limonene, 50. 2-hexanol, 51. 1-pentanol, 52. 3-methyl-2-buten-1-ol, 53. *n*-hexanol, 54. *trans*-3-hexen-1-ol, 55. *trans*-2-hexen-1-ol, 56. 2-ethyl hexanol, 57. 2,5-dimethylcyclohexanol, 58. 2,5-dimethylcyclohexanol, 59. 2-methylthio ethanol, 60. linalool, 61. (+)-isomenthol, 62. nona-3,6-dien-1-ol, 63. geraniol, 64. guaiacol, 65. benzyl alcohol, 66. phenethyl alcohol, 67. 4-ethyl-2-methoxy phenol, 68. *m*-cresol, 69. eugenol. 70. *n*-decane, 71. 3-methyl-pentan-2-one, 72. ethyl butanoate, 73. 3-hexanone, 74. ethyl isopentanoate, 75. 2-hexanone, 76. 4-heptanone, 77. 2-heptanone, 78. butyl butanoate, 79. ethyl hexanoic acid, 80. isopentyl butanoate, 81. 2-octanone, 82. isoamyl isopentanoate, 83. *n*-tridecane, 84. 2-nonanone, 85. acetophenone, 86. *γ*-decanolactone, 87. methyl anthranilate.

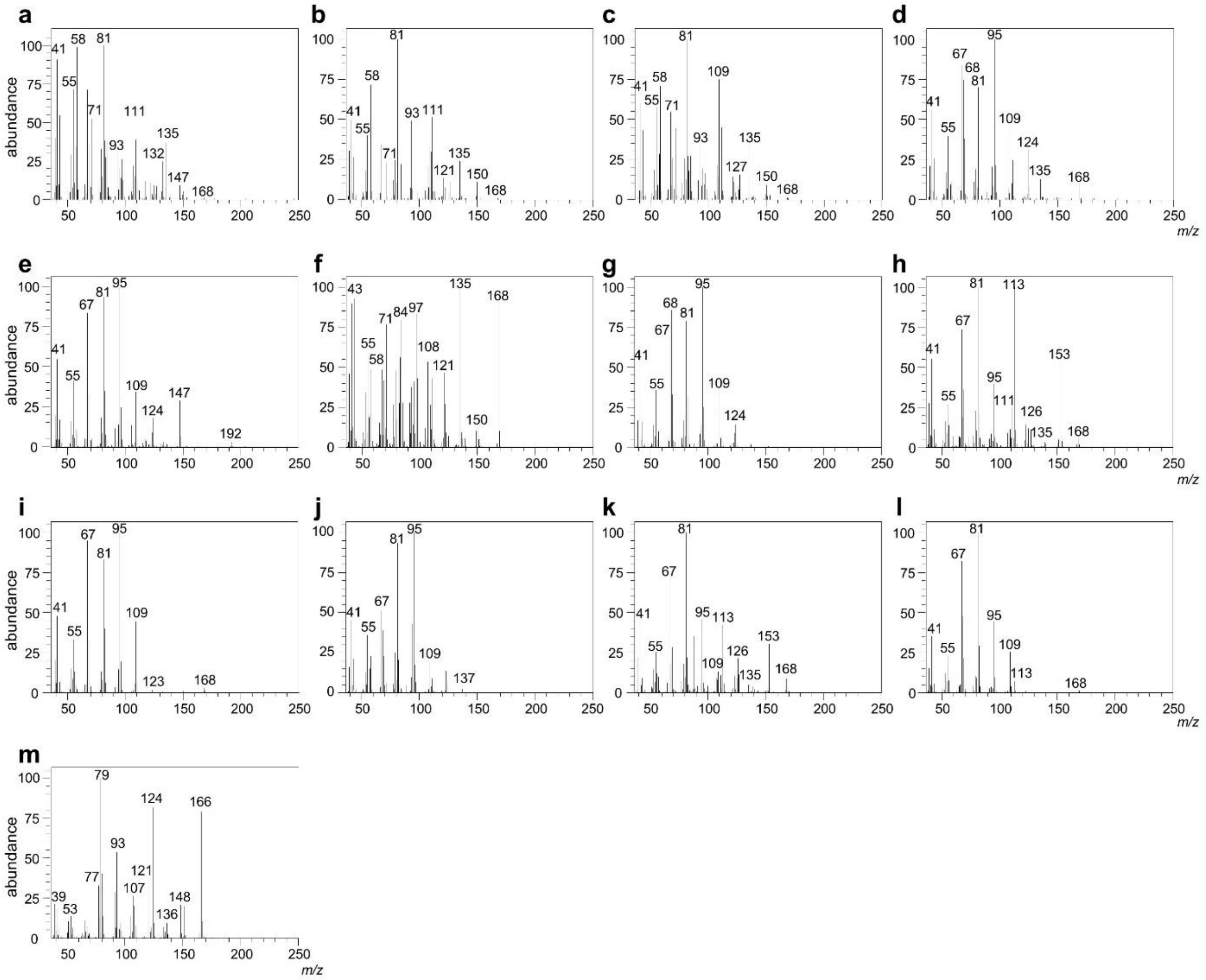

Supplementary Fig. 2. Mass spectra of four iridoids and nine candidate iridoids in the silver vine extract, related to Fig. 4.
Characters (a–m) are corresponding to GC-MS peaks in Figure 4A. b and c. candidates of *trans-trans* iridodial, f. *cis-trans* nepetalactol, h. isodihydronepetalactone, i. isoiridomyrmecin, k. dihydronepetalactone, l. candidate of isoepiiridomyrmecin, m. isoneonepetalactone, others, unknown candidate iridoids.
